# Atypical acute disseminated encephalomyelitis with systemic inflammation after a first dose of AztraZaneca COVID-19 vaccine. A case report

**DOI:** 10.3389/fneur.2022.995875

**Published:** 2022-08-29

**Authors:** Laure Bastide, Gaetano Perrotta, Valentina Lolli, Céline Mathey, Ortensa-Irina Vierasu, Serge Goldman, Frédéric Vandergheynst

**Affiliations:** ^1^Department of Neurology, CUB Hôpital Erasme, Université Libre de Bruxelles, Brussels, Belgium; ^2^Department of Radiology, CUB Hôpital Erasme, Université Libre de Bruxelles, Brussels, Belgium; ^3^Department of Nuclear Medicine, CUB Hôpital Erasme, Université Libre de Bruxelles, Brussels, Belgium; ^4^Department of Internal Medicine, CUB Hôpital Erasme, Université Libre de Bruxelles, Brussels, Belgium

**Keywords:** acute disseminated encephalomyelitis (ADEM), COVID-19, vaccination, systemic inflammation, fluorodeoxyglucose positron emission tomography with computed tomography (FDG-PET/CT)

## Abstract

**Background:**

Only a few cases of acute disseminated encephalomyelitis (ADEM) following coronavirus disease 19 (COVID-19) vaccination have been described since the beginning of the vaccination campaign.

**Results:**

Here we report the first case of central nervous system (CNS) demyelination with systemic inflammatory findings on whole body 19-fluorodeoxyglucose positron emission tomography with computed tomography (FDG-PET/CT) following the ChAdOx1 nCoV-19 vaccine.

**Conclusions:**

Clinicians should stay aware of potential new adverse events after immunization.

## Introduction

Since the beginning of the pandemic, vaccines were produced in record time. Real-world studies indicated an excellent safety profile. Despite these studies, the scientific community must stay aware of rare but severe complications and report them. This allows more accuracy of the real-world safety profile of the vaccine. We can take appropriate measures, as we did with the AztraZaneca vaccine (ChAdOx1 nCoV-19) and its thromboembolic complications (1). The ChAdOx1 nCoV-19 is a vaccine based on a recombinant adenoviral vector encoding the spike protein of SARS-CoV-2 (2). Acute disseminated encephalomyelitis (ADEM) is an immune-mediated inflammatory disorder of the central nervous system (CNS) that occurs after an antigenic challenge. The post-vaccine etiology represents 5% of all ADEM cases and the annual incidence of ADEM ranges from 1 to 10 per million (3). Here we report the first case of central nervous system (CNS) demyelination with systemic inflammatory findings on whole body 19-fluorodeoxyglucose positron emission tomography with computed tomography (FDG-PET/CT) following the ChAdOx1 nCoV-19 vaccine.

## Case report

A previously healthy 49-year-old female received her first dose of ChAdOx1 nCoV-19 vaccine. She experienced mild flu-like symptoms during the following 48 h. One week later, the patient presented another episode of flu-like symptoms with fever, fatigue, neck pain, followed over the next few days by rapidly progressive sensitive symptoms including paresthesia in both legs, up to the chest, Lhermitte's phenomenon and sphincter dysfunction. In April, the patient came to the neurological consultation at another hospital. During the examination a hypoesthesia with a thoracic (Th) 8 level was noticed with a sensory ataxia. A full spine magnetic resonance imaging (MRI) was normal but somatosensory evoked potentials (SSEPs) showed abnormal conduction above the sensory decussation in the lower brainstem. Four weeks later, the patient came to our neurological outpatient clinic. Her symptoms had worsened with sensory symptoms now involving her hands, worsening sensory ataxia and of sphincter dysfunction. Her neurological examination showed normal strength, hypoesthesia to all modalities with a Th 8 level, absent plantar response, impaired tandem walking and the presence of a Romberg sign.

**Figure 1:**
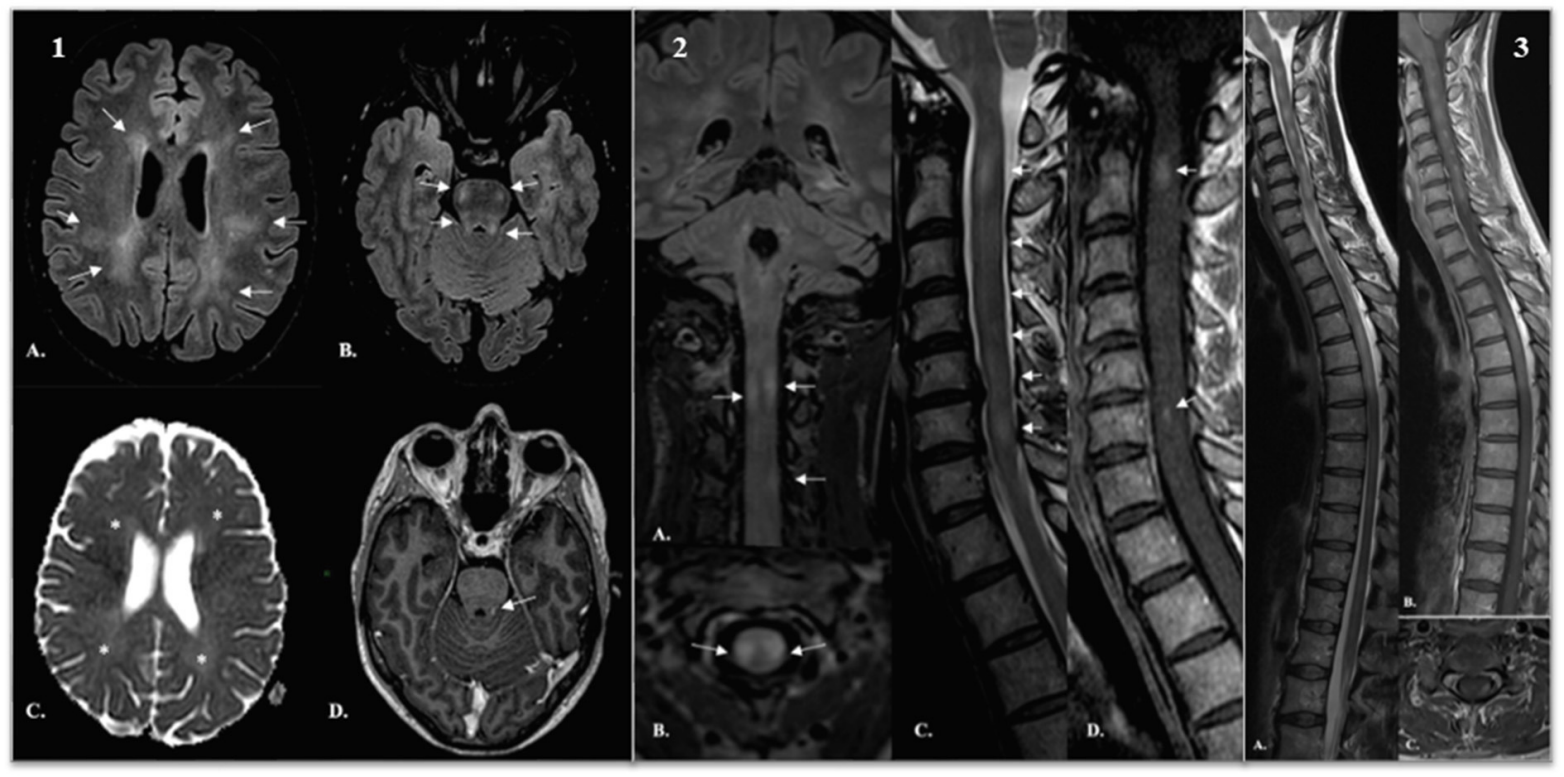
Part 1: (A,B) Axial T2 FLAIR-weighted images demonstrated extensive, asymmetric involvement of periventricular and deep white matter [arrows in (A)]. Smaller lesions were observed in the ponto-mesencephalic tegmentum, superior and middle cerebellar peduncles [arrows in (B)]. (C) Lesions were mildly hyperintense on ADC cartography, revealing increased diffusivity (*). (D) A small focus of contrast enhancement was demonstrated in the left superior cerebellar peduncle (arrow). Part 2: (A–C) Reformatted coronal (A) and axial (B) T2 FLAIR-weighted images and sagittal T2-weighted images and sagittal T2 weighted image (C) reveal multiple short-segment hyperintensities (arrows). Lesions are asymmetric and excentrically located and involve both white and gray matter. Signal intensity is variable, from midly to markedly increased. (D) Sagittal post-gadolinium T1-weighted image shows scattered foci of enhancement (arrows). Part 3: (A–C) Sagittal T2 (A) and post-contrast sagittal (B) and axial (C) T1-weighted images demonstrate progression of disease. We found lesions on the entire spinal cord. FLAIR, fluid attenuated inversion recovery; ADC, apparent diffusion coefficient.

An MRI of the brain was obtained and revealed large, ill-defined T2 fluid attenuated inversion recovery (FLAIR) hyperintensities of periventricular and deep white matter, along with smaller lesions infratentorially (Figure 1, part1). Subcortical U fibers were spared, and so were the cortex and deep gray matter. Lesions showed mildly increased diffusivity and were mostly non-enhancing. They exerted no mass effect. No meningeal enhancement was noted. MRI of the spinal cord revealed the appearance of numerous contiguous short-segment cervical and thoracic lesions, showing variably increased T2 signal intensity and contrast enhancement (Figure 1, part 2). The spinal cord was moderately swollen. Nerve conductive studies were normal. A lumbar puncture showed a mild pleocytosis with 8 white blood cells, elevated protein levels (101 mg/dL), normal IgG/albumin index and identical oligoclonal bands in the cerebrospinal fluid (CSF) and serum (type 4 pattern). Based on the clinical history and the radiological aspects an inflammatory origin was retained. Our differential diagnosis workup was mainly focused on an infectious or an auto-immune causes. An infectious panel was negative. An exhaustive blood investigation was done with the intention to exclude auto-immune systemic diseases, no relevant findings were found. A screening for antibodies targeting antigens associated with demyelinating disorders of the CNS (MOG antibody disease and NMO spectrum) remained negative (Table 1).

**Table 1:** Clinical evolution and complementary assessments done during patient follow-up.

**Temporality**	**Neurological examination**	**Laboratory investigations**	**Cerebrospinal fluid investigations**	**MRIs**	**Others**	**Treatment**
April 2021	Hypoesthesia with a Th8 level Lhermitte phenomenon Sensitive ataxia Sphincter dysfunction.	Thyroid, hepatic, hematologic and renal functions normal.	None.	Normal spinal MRI.	SSEPs: asymetric conduction of the somesthetic influx with a subcortical but supralemniscal level.	
May and June 2021	As above but with decreased pallesthesia and absent plantar response.	serum protein electrophoresis, vitamins, angiotensin converted enzyme, erythrocyte sedimentation speed, microbiological studies (including Tuberculosis – QuantiFERON blood test, HAV, HBV, EBV, CMV, HIV, HSV, Syphilis, Borrelia, Toxoplasma, JC virus, SARS-CoV-2), screening for antibodies targeting antigens associated with demyelinating disorders of the CNS (MOG, AQP4) and other auto-immune disorders (ANA, ANCA) remained negative.	8 WBC, protein level at 101 mg/dl, normal IgG/Albumin Index, OCBs identical in CSF and serum. Negative infectious panel.	Extensive, asymmetric involvement of periventricular and deep white matter. Smaller lesions were observed in the ponto-mesencephalic tegmentum, superior and middle cerebellar peduncles. Lesions were mildly hyperintense on ADC cartography, revealing increased diffusivity. A small focus of contrast enhancement was demonstrated in the left superior cerebellar peduncle.	Normal nerve conductive studies.	IV MP 1 gr/day, for 5 days.
July 2021	Paraparesis 2/5 in the right leg and 3/5 in the left leg. Apallesthesia up to iliac crests. Sensory level at Th5 level. Need walking aids	Negative MOG and AQP4 antibodies. Negative ANA and ANCA.		Numerous contiguous short-segment cervical and thoracic lesions, showing variably increased T2 signal intensity and contrast enhancement.		5 sessions of Therapeutic Plasma Exchange.
August 2021	Weakness worsened after an improvement.	Normal thyroid hormone level and autoantibodies. Negative ANA and ANCA Negative MOG antibody.	2 WBC, protein level at 95mg/dl, normal IgG/Albumin Index, OCBs identical in CSF and serum. Negative infectious panel.	Increase in the number and size of spinal cord lesions and the appearance of new foci of contrast enhancement. Brain findings were unchanged.	FDG PET-CT: increased glucose uptake in the thyroid, the pulmonary nodules, the thoracic aorta walls, the lumbar spinous processes and the whole spinal cord. Normal thyroid echography.	Rituximab 1 gr IV in 2 times at 15 days and another course of IV MP.
November 2021	Paraparesis 3+/5 in the right leg and 4+/5 in the left leg. Sensory level at Th12. Few steps without help.			Brain and spinal MRIs stable or regression of the most enhanced lesions.	FDG PET-CT: thyroid and pulmonary uptake disappeared or decreased, new uptake in scapular and pelvic belts, ischiatic, and great trochanters.	

Th, thoracic; SSEPs, somatosensory evoked potentials; WBC, white blood cells, OCBs, oligoclonal bands; CSF, cerebrospinal fluid; MRI, magnetic resonance imaging; ADC, apparent diffusion coefficient; DWI, diffusion-weighted imaging; IV, intravenous; MP, methylprednisolone; FDG PET-CT,fluorodeoxyglucose positron emission tomography with computed tomography (FDG-PET/CT).

Based on the exclusion of CNS infection or other autoimmune disorders, the diagnosis of atypical ADEM was made. The patient was treated with an intravenous course of methylprednisolone (1 g/day for 5 days). Her condition stabilized and she was transferred to a rehabilitation center.

Three weeks after discharge, she was readmitted because of a clinical deterioration. Neurological evaluation showed a new paraparesis, evaluated at 2/5 in the right leg and 3/5 in the left leg, complete loss of pallesthesia up to the iliac crests, a sensory Th 5 level, and a severe sensory ataxia requiring walking aids.

Six weeks afterwards, on July 14, an MRI showed an increase in the number and size of spinal cord lesions and the appearance of new foci of contrast enhancement (Figure 1, part 3). Brain findings were unchanged. She was treated with 5 sessions of plasma exchange. She improved and was discharged again to a rehabilitation center. Three weeks later, her weakness worsened. A new MRI showed there were new enhancing lesions in the brain stem and cervical spinal cord.

**Figure 2:**
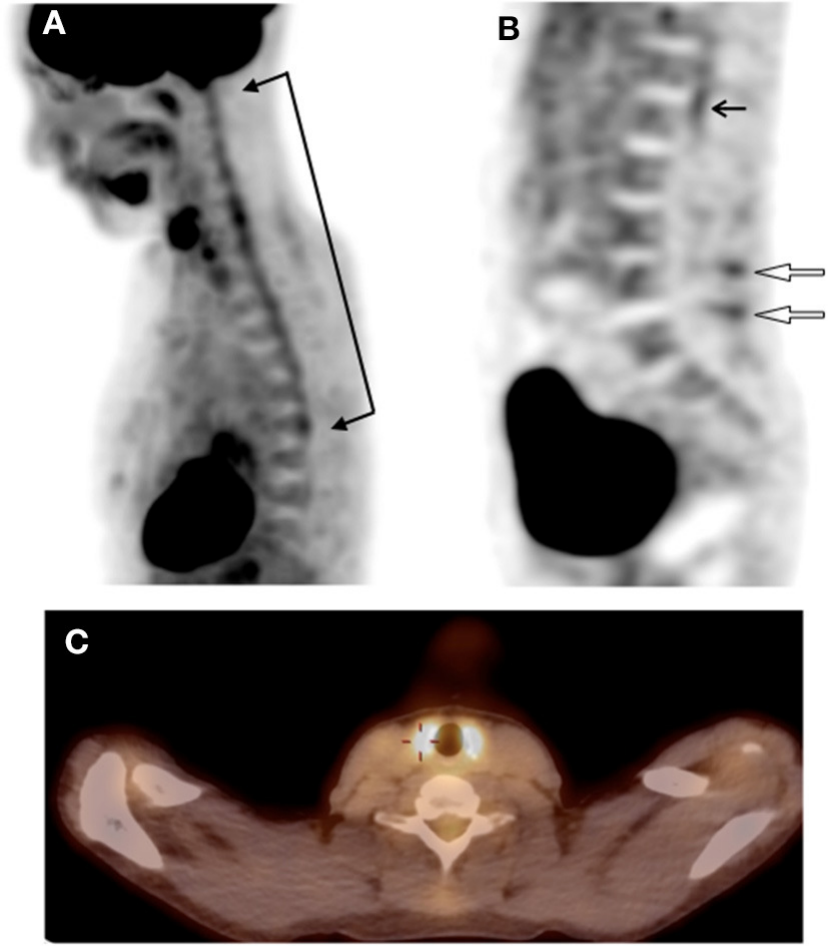
**(A)** sagittal view of FDG-PET/CT showing hypermetabolism of the spinal cord at the cervicothoracic level (between arrows). **(B)** sagittal view of FDG-PET/CT showing hypermetabolism of the spinal cord at the lumbosacral level (black arrow) and an interspinous hypermetabolism at two level of the lumbar spine (open arrows). **(C)** fused FDG-PET/CT image on the transverse plane showing a marked and diffuse hypermetabolism of the thyroid gland. FDG-PET/CT, whole body 19-fluorodeoxyglucose positron emission tomography with computed tomography.

Because of the atypical course of the disease, the diagnosis of ADEM was reconsidered. Whole body fluorodeoxyglucose positron emission tomography with computed tomography (FDG-PET/CT) was obtained with the aim of excluding systemic inflammation, namely sarcoidosis despite negative biological markers. Results revealed increased glucose uptake not only in the spinal cord but also in the thyroid, the thoracic aorta walls and the lumbar spinous processes (Figure 2).

She did not have any complaints about osteo-articular or vascular systems. Further thyroid testing with echography, hormone levels and autoantibodies were normal. She received IV rituximab (1 g and another after 15 days) and another course of IV methylprednisolone (1 g/day for 5 days). Over the next 2 months, she progressively improved. At last follow-up, strength in her right and left lower limbs was evaluated at 3+/5 and 4+/5, respectively, the sensory level had dropped to the level of Th12 and she could take a few steps without aid. Another MRI showed stability or regression of most lesions.

On repeat whole body FDG-PET/CT (13 weeks after the first one), thyroid uptake had disappeared but other regions' abnormal uptake had decreased and new areas of increased uptake had appeared at the level of the scapular and pelvic girdles, ischiatic and great trochanters.

## Discussion

This case report raises two important points: the association between ChAdOx1 nCoV-19 and ADEM and the meaning of incidental findings in the FDG PET-CT.

A review of the SARS-CoV-2 vaccine and ADEM literature showed 13 reported cases of ADEM following the administration of a COVID-19 vaccine, which are summarized in Table 2 (4–14).

**Table 2:** Clinical and demographic characteristics of the 6 cases reporting with an ADEM post COVID vaccine.

**Authors**	**Age/gender**	**Vaccine**	**Time of onset**	**Clinical picture**	**CSF/laboratory investigations**	**MRI**	**Treatment**	**Outcome/follow-up**
Cao et al. (4)	24y/F	Sinovac, inactivated vaccine	1st dose, 14 d after	Memory decline, headache, low-grade fever, muscle stiffness, extremity weakness, and reduced appetite. GTCs after one week.	Negative anti-AQP4, anti-MOG antibodies, vasculitis, OCBs.	Brain lesions, no enhancement.	IVIG 20 g/d for 5 d.	No recurrences. Marked improvement. Complete resolution of MRI lesions. No seizures, 30d.
Ozgen Kenangil et al. (5)	46y/F	Sinovac, inactivated vaccine	2nd dose, 30d after	GTCs.	Negative OCBs.	Brain lesions, no enhancement.	IV MP 1 g/d for 7d.	No recurrences. Stable. No seizure recurrence.
Raknuzzaman et al. (6)	55y/M	mRNA-based vaccine	1st dose, 21d after	Headache, somnolence, delirium and GTCs.	Normal ESR.	Brain lesions.	IV MP 1 g/d for 5d followed by oral tapering steroids.	No recurrences. Improvement of MRI lesions and fully recovered, 30d.
Vogrig et al. (7)	56y/F	Pfizer-BioNTech COVID-19, mRNA-based vaccine	1st dose, 14d after	Malaise, chills, without fever, followed by unsteady gait, clumsiness of left arm.	Negative: anti-AQP4, anti-MOG antibodies, vasculitis, OCBs.	Brain lesions, no enhancement.	Prednisone 75mg q.d. with gradual tapering.	No recurrences. Partial improvement, 50d.
Kania et al. (8)	19y/F	Moderna, mRNA-based vaccine	1st dose, 14d after	Severe headache, fever, back and neck pain, nausea, vomiting, urinary retention.	Negative: anti-AQP4, anti-MOG antibodies, OCBs.	Brain and medullar lesions with enhancement.	IV MP and TPE (stopped because of allergic reaction)	No recurrences. Mild headache, 40d.
Rinaldi et al. (9)	45y/M	ChAdOx1 nCoV-19, viral vector	1st dose, 12d after	Numbness of all the upper limbs, trunk, and legs and progressive reduced visual acuity, dysarthria, dysphagia, clumsy right-hand movements and urge incontinence.	Negative: anti-AQP4, anti-MOG antibodies, ANA, ESR, OCBs.	Brain and medullar lesions with enhancement.	IV MP 1 g/d followed by oral prednisolone.	No recurrences. Complete recovery, 4months.
Permezel et al. (10)	63y/M	ChAdOx1 nCoV-19, viral vector	1st dose, 12d after	Vertigo, fatigue, declining cognition, disorientation and impaired attention.	Negative: anti-AQP4, anti-MOG, anti-neuronal, anti-NMDAR, anti-LGI-1 and anti-forantivoltage gated K+ channel antibodies. OCBs positive.	Brain and medullar lesions without enhancement.	IV MP 1 g/d 5d followed by TPE.	Death 20d after hospitalization.
Shimizu et al. (11)	88y/F	Pfizer-BioNTech COVID-19, mRNA-based vaccine	2nd dose, 29d after	Impaired consciousness and gaze-evoked nystagmus.	Negative: anti-onconeuronal, anti-ganglioside antinuclear, autoimmune vasculitis and MBP antibodies, OCBs.	Brain lesions without enhancement.	IV MP 1 g/d 3d.	Clinical and MRI improvement after 66d.
Al-Quliti et al. (12)	56y/F	ChAdOx1 nCoV-19, viral vector	1st dose, 10d after	Paraparesis and slurred speech.	/	Brain lesions	IV steroids.	Clinical improvement.
Nagaratnam et al. (13)	36y/F	ChAdOx1 nCoV-19, viral vector	1^st^ dose, 14d after	Bilateral visual impairment and headache. Pseudo relapse 15d after the onset.	Negative: anti-AQP4, anti-MOG, ANCA, ANA. OCBs positive.	Brain lesions with enhancement and no spinal lesion.	Two courses of IV MP 1 g/d 3d with a prednisolone tapering plan.	Clinical resolution and MRI improvement at 42d.
Ancau et al. (14)	61y/M	ChAdOx1 nCoV-19, viral vector	1^st^ dose, 2d after	Fever, headache, apathy and then unconsciousness and GS.	Negative: anti-AQP4, anti-MOG, ANA, ANCA, anti-neuronal and paraneoplasic antibodies, OCBs.	Brain lesions with hemorrhages.	IV MP 1 g/d 5d followed by TPE with concomitant oral MP.	MRI improvement at 5d and vegetative state after 98d.
Ancau et al. (14)	25y/F	ChAdOx1 nCoV-19, viral vector	1^st^ dose, 9d after	Cephalalgia, thoracic back pain, paraplegic syndrome with Anesthesia below dermatome Th6, sphincter dysfunction.	Negative: anti-AQP4, anti-MOG, ANA, ANCA, anti-neuronal and paraneoplastic antibodies, OCBs.	Brain and spinal lesions with enhancement and hemorrhages.	IV MP 1 g/d 5d followed by TPE with concomitant oral MP	Clinical improvement of sensory symptoms at 42d.
Ancau et al. (14)	55y/F	ChAdOx1 nCoV-19, viral vector	1^st^ dose, 9d after	Nausea, dizziness and meningism, worsened to severe spastic tetraparesis and coma.	Negative: anti-AQP4, anti-MOG, ANA, ANCA, anti-neuronal and paraneoplastic antibodies, OCBs.	Brain lesions with hemorrhages.	IV MP 1 g/d 5d.	Death
Our case report	49y/F	ChAdOx1 nCoV-19, viral vector	1st dose, 7d after	Neck pain, fatigue, fever, partial transverse myelitis and sphincter dysfunction Two recurrences.	Negative: AQP4, MOG antibodies, ANA, ANCA, ESR, OCBs.	Brain and medullar lesions with enhancement.	IV MP 1 g/d during 5d, TPE 5 sessions, Rituximab 2gr and IV MP 1 g/d during 5d.	Mild improvement, 9 months.

CSF, Cerebrospinal fluid; MRI, magnetic resonance imaging; FDG PET-CT, F-fluorodeoxyglucose positron emission tomography with computed tomography; Y, years; F,Female; M, Male; d, days, GTCs, Generalized Tonico-Clonic seizures; OCBs, OligoClonal Bands; ESR, Erythrocyte Sedimentation Rate; IV, Intravenous; IG, Immunoglobulins; MP, Methylprednisolone; TPE, Therapeutic Plasma Exchange; Th, Thoracic.

In comparison to prior cases except maybe one who had a pseudo relapse (14), our patient had a more protracted course, which evolved in two subsequent worsening phases until improvement 8 months later. These phases occurred each time after treatment cessation and there was no relapse after the symptomatic nadir which occurred in August (Table 1). Therefore, we conclude that these recurrences are part of the same monophasic course.

Also, the MRI evolution of the lesions is atypical for several reasons: the sub-acute evolution (longer than 3 months), the discordance between brain and spinal cord lesions in terms of how they evolved and their aspects, and the limited resolution on the last MRI after 7 months of follow-up. As some studies have described, some lesions could take up to 18 months to disappear (15) or persisted on follow-up imaging (16). We did have the information of the MRI evolution from only 3 previously reported cases as shown in Table 2: one with a complete resolution in 1 month (4) and the other two with a partial resolution at follow-up of 30 and 66d (6, 11). We retained the diagnosis of ADEM according to Sejvar et al. (17) but determined it atypical because of these particular findings.

It is the first reported case of post-ChAdOx1 nCoV-19 vaccination ADEM in which FDG-PET/CT was performed. The observed pulmonary nodules' hypercaption were very small (<5 mm) with a reduction of the glucose uptake at the FDG-PET/CT control. A basic control will be performed at one year with a CT.

The increased glucose uptake observed in the thyroid on the first FDG-PET/CT is difficult to interpret in our clinical setting. Mild FDG uptake by the thyroid is likely physiological and a normal variant but moderate-to-intense diffuse uptake is usually associated with elevated TSH, thyroiditis, hyperthyroidism or Graves' disease (18). One interesting study reported aortic and thyroid unexpected hypermetabolism without clinical relevance in a cohort of patients with anti-neutrophil cytoplasmic antibodies-associated vasculitis (19). In our case the complementary analysis and also the control FDG-PET/CT were normal, leading to the conclusion that the initial thyroid finding had no clinical relevance.

The increased uptake of the thoracic aorta and the lumbar spinous processes interspaces associated with the increased uptake of the scapular and pelvic girdles, ischiatic and great trochanters in the second FDG-PET/CT raised the question of polymyalgia rheumatic associated with a giant cell arteritis (PMR-GCA) diagnosis. Again, in our case we did not have any clinical correlation and our patient is substantially younger (40 years old) than the median age (70 years old) of diagnosis for this PMR-GCA entity (20). We did not find any description in the literature of the association of ADEM with vasculitis, in particular giant-cell arteritis. Large-vessel vasculitis is not classically associated with extensive myelitis. We only found a case report of NMO spectrum disorder which is a demyelinating auto-immune disease of the CNS, associated with Takayasu arteritis (21). The possibility of CNS and systemic vasculitis, triggered by the vaccination in our case, should be raised. Recent literature reports cases of vasculitis as cutaneous vasculitis (22), hypersensitivity angiitis, IgA vasculitis (23) and ANCA-associated vasculitis (24) following ChAdOx1 nCoV-19 vaccine and one case of eosinophilic granulomatosis with polyangiitis after the Moderna vaccine (25). We also found one reported case of CNS vasculitis following BNT162b2, Pfizer/BioN-Tech vaccine (26) but without FDG-PET/CT done. Vasculitis was described as a complication during COVID-19 because of direct endothelial damage (27) and ChAdOx1 nCoV-19 vaccine is associated with immune thrombosis and thrombocytopenia. To date, current data do not strongly support a causative link between vaccination and most of vasculitis (28). The hypothesis of two autoimmune disorders coexistence' rather than a large-vessel vasculitis with CNS involvement could also be raised and it is a situation already described in the literature (29, 30). In our case, the lack of clinical corresponding symptoms to the FDG-PET/CT findings does not allow to confirm a specific diagnosis. For all these reasons we will remain for now with the diagnosis of atypical ADEM with systemic inflammation without a clear diagnosis.

## Conclusions

We report the first case of post-ChAdOx1 nCoV-19 vaccination atypical ADEM with incidental findings on the FDG-PET/CT consistent with a large-vessel vasculitis, in particular GCA given the hypermetabolism of scapular and pelvic girdles and typical of polymyalgia rheumatica. Their relevance remains debatable at this stage given the lack of corresponding symptoms. Clinicians should stay aware of potential new adverse events after immunization.

## Data availability statement

The original contributions presented in the study are included in the article/supplementary material, further inquiries can be directed to the corresponding author/s.

## Ethics statement

The studies involving human participants were reviewed and approved by Comité d'Ethique de l'Hopital Erasme—CUB. The patients/participants provided their written informed consent to participate in this study. Written informed consent was obtained from the individual(s) for the publication of any potentially identifiable images or data included in this article.

## Author contributions

LB wrote the first draft of the manuscript. VL, SG, MC, and VO-I wrote sections of the manuscript. All authors contributed to manuscript revision, read, and approved the submitted version.

## Conflict of interest

The authors declare that the research was conducted in the absence of any commercial or financial relationships that could be construed as a potential conflict of interest.

## Publisher's note

All claims expressed in this article are solely those of the authors and do not necessarily represent those of their affiliated organizations, or those of the publisher, the editors and the reviewers. Any product that may be evaluated in this article, or claim that may be made by its manufacturer, is not guaranteed or endorsed by the publisher.

## Abbreviations

ADEM: acute disseminated encephalomyelitis
Coronavirus disease 19: COVID-19
CNS: central nervous system
FDG-PET/CT: whole body 19-fluorodeoxyglucose positron emission tomography with computed tomography
Th: thoracic
MRI: magnetic resonance imaging
SSEPs: somatosensory evoked potentials
FLAIR: fluid attenuated inversion recovery
ADC, apparent diffusion coefficient PMR-GCA: polymyalgia rheumatic associated with a giant cell arteritis.

## References

[1] Greinacher A Thiele T Warkentin TE Weisser K Paul A. Kyrle, Eichinger S. Thrombotic thrombocytopenia after ChAdOx1 nCov-19 vaccination. N Engl J Med. (2021) 384:2092–101. 10.1056/NEJMoa2104840 33835769 PMC8095372

[2] Voysey M Clemens SAC Madhi SA. Safety and efficacy of the ChAdOx1 nCoV-19 vaccine (AZD1222) against SARS-CoV-2: an interim analysis of four randomised controlled trials in Brazil, South Africa, and the UK. Lancet. (2021) 397:99–111. 10.1016/S0140-6736(20)32661-1 33306989 PMC7723445

[3] Pellegrino P Carnovale C Perrone V. Acute disseminated encephalomyelitis onset: evaluation based on vaccine adverse events reporting systems. PLoS ONE. (2013) 8:e77766. 10.1371/journal.pone.0077766 24147076 PMC3797690

[4] Cao L Ren L. Acute disseminated encephalomyelitis after severe acute respiratory syndrome coronavirus 2 vaccination: a case report. Acta Neurol Belg. (2021) 1:1–3. 10.1007/s13760-021-01608-2 33527327 PMC7849959

[5] Ozgen Kenangil G Ari BC Guler C Demir MK. Acute disseminated encephalomyelitis-like presentation after an inactivated coronavirus vaccine. Acta Neurol Belg. (2021) 121:1089–91. 10.1007/s13760-021-01699-x 34018145 PMC8136261

[6] Raknuzzaman M Jannaty T Hossain MB Saha B Dey SK Shahidullah M. Post Covid19 vaccination acute disseminated encephalomyelitis: a case report in Bangladesh. Int J Med Sci Clin Res Stud. (2021) 1:31–6.

[7] Vogrig A Janes F Gigli GL Curcio F Negro ID D'Agostini S. Acute disseminated encephalomyelitis after SARS-CoV-2 vaccination. Clin Neurol Neurosurg. (2021) 208:106839. 10.1016/j.clineuro.2021.106839 34325334 PMC8294707

[8] Kania K Ambrosius W Tokarz Kupczyk E Kozubski W. Acute disseminated encephalomyelitis in a patient vaccinated against SARS-CoV-2. Ann Clin Transl Neurol. (2021) 8:2000–3. 10.1002/acn3.51447 34480527 PMC8528462

[9] Rinaldi V Bellucci G Romano A Bozzao A Salvetti M. ADEM after ChAdOx1 nCoV-19 vaccine: A case report. Mult Scler. (2021) 28:1151–4. 10.1177/13524585211040222 34590902

[10] Permezel F Borojevic B Lau S de Boer HH. Acute disseminated encephalomyelitis (ADEM) following recent Oxford/AstraZeneca COVID-19 vaccination. Forensic Sci Med Pathol. (2022) 18:74–9. 10.1007/s12024-021-00440-7 34735684 PMC8567127

[11] Shimizu M Ogaki K Nakamura R Kado E Nakajima S Kurita N. An 88-year-old woman with acute disseminated encephalomyelitis following messenger ribonucleic acid-based COVID-19 vaccination. eNeurologicalSci. (2021) 25:100381. 10.1016/j.ensci.2021.100381 34841097 PMC8605821

[12] Al-Quliti K Qureshi A Quadri M Abdulhameed B Alanazi A Alhujeily R. Acute demyelinating encephalomyelitis post-COVID-19 vaccination: a case report and literature review. Diseases. (2022) 10:13. 10.3390/diseases10010013 35225865 PMC8884009

[13] Nagaratnam SA Ferdi AC Leaney J Lee RLK Hwang YT Heard R. Acute disseminated encephalomyelitis with bilateral optic neuritis following ChAdOx1 COVID-19 vaccination. BMC Neurol. (2022) 22:54. 10.1186/s12883-022-02575-8 35151258 PMC8840677

[14] Ancau M Liesche-Starnecker F Niederschweiberer J. Case series: acute hemorrhagic encephalomyelitis after SARS-CoV-2 vaccination. Front Neurol. (2021) 12:820049. 10.3389/fneur.2021.820049 35185757 PMC8847228

[15] Honkaniemi J Dastidar P Kähärä V Haapasalo H. Delayed MR imaging changes in acute disseminated encephalomyelitis. AJNR Am J Neuroradiol. (2001) 22:1117–24. 11415907 PMC7974795

[16] van der Knaap MS Valk J eds. Acute disseminated encephalomyelitis and acute hemorrhagic encephalomyelitis. In: Magnetic Resonance of Myelination and Myelin Disorders. (2005). Berlin: Springer. p. 604–15. 10.1007/3-540-27660-2_80

[17] Sejvar JJ Kohl KS Bilynsky R. Encephalitis, myelitis, and acute disseminated encephalomyelitis (ADEM): case definitions and guidelines for collection, analysis, and presentation of immunization safety data. Vaccine. (2007) 25:5771–92. 10.1016/j.vaccine.2007.04.060 17570566

[18] Liu Y Ghesani NV Zuckier LS. Physiology and pathophysiology of incidental findings detected on FDG-PET scintigraphy. Semin Nucl Med. (2010) 40:294–315. 10.1053/j.semnuclmed.2010.02.002 20513451

[19] Kemna MJ Vandergheynst F Vöö S Blocklet D Nguyen T Timmermans SAMEG. Positron emission tomography scanning in anti-neutrophil cytoplasmic antibodies-associated vasculitis. Medicine (Baltimore). (2015) 94:e747. 10.1097/MD.0000000000000747 25997040 PMC4602883

[20] Slart Slart RHJA; Writing group; Reviewer group; Members of EANM Cardiovascular; Members of EANM Infection & Inflammation; Members of Committees SNMMI Cardiovascular . FDG-PET/CT(A) imaging in large vessel vasculitis and polymyalgia rheumatica: joint procedural recommendation of the EANM, SNMMI, and the PET Interest Group (PIG), and endorsed by the ASNC. Eur J Nucl Med Mol Imaging. (2018) 45:1250–69. 10.1007/s00259-018-3973-8 29637252 PMC5954002

[21] Lamartine S. Monteiro M, Lascano AM, Meunier Carus Vincent N, Seebach JD, Lalive PH, Gschwind M. AQP4 antibody-positive NMO spectrum disorder associated with *Takayasu arteritis.* J Neurol Sci. (2019) 396:130–2. 10.1016/j.jns.2018.11.016 30453208

[22] Cavalli G Colafrancesco S De Luca G Rizzo N Priori R Conti F. Cutaneous vasculitis following COVID-19 vaccination. Lancet Rheumatol. (2021) 3:e743–4. 10.1016/S2665-9913(21)00309-X 34611627 PMC8483649

[23] Pottegård A Lund LC Karlstad Ø Dahl J Andersen M Hallas J. Arterial events, venous thromboembolism, thrombocytopenia, and bleeding after vaccination with Oxford-AstraZeneca ChAdOx1-S in Denmark and Norway: population based cohort study. BMJ. (2021) 373:n1114. 10.1136/bmj.n1114 33952445 PMC8097496

[24] Villa M Díaz-Crespo F Pérez de José A Verdalles Ú Verde E Almeida Ruiz F. A case of ANCA-associated vasculitis after AZD1222 (Oxford-AstraZeneca) SARS-CoV-2 vaccination: casualty or causality? Kidney Int. (2021) 100:937–8. 10.1016/j.kint.2021.07.026 34416184 PMC8372491

[25] Ibrahim H Alkhatib A Meysami A. Eosinophilic Granulomatosis With Polyangiitis Diagnosed in an Elderly Female After the Second Dose of mRNA Vaccine Against COVID-19. Cureus. (2022) 14:e21176. 10.7759/cureus.21176 35165624 PMC8831231

[26] Takeyama R Fukuda K Kouzaki Y. Intracerebral hemorrhage due to vasculitis following COVID-19 vaccination: a case report. Acta Neurochir (Wien). (2022) 164:543–7. 10.1007/s00701-021-05038-0 34783899 PMC8594320

[27] McGonagle D Bridgewood C Ramanan AV Meaney JFM Watad A. COVID-19 vasculitis and novel vasculitis mimics. Lancet Rheumatol. (2021) 3:e224–33. 10.1016/S2665-9913(20)30420-3 33521655 PMC7832717

[28] Bonetto C Trotta F Felicetti P. Vasculitis as an adverse event following immunization - Systematic literature review. Vaccine. (2016) 34:6641–51. 10.1016/j.vaccine.2015.09.026 26398442

[29] Wingerchuk DM Weinshenker BG. The emerging relationship between neuromyelitis optica and systemic rheumatologic autoimmune disease. Mult Scler. (2012) 18:5–10. 10.1177/1352458511431077 22146604

[30] Zhang B Zhong Y Wang Y. Neuromyelitis optica spectrum disorders without and with autoimmune diseases. BMC Neurol. (2014) 14:162. 10.1186/s12883-014-0162-7 25135481 PMC4236652

